# “Pearls”: A New Type of Open-Access Educational Resource

**DOI:** 10.1371/journal.ppat.1000499

**Published:** 2009-06-26

**Authors:** Hiten D. Madhani, Kasturi Haldar, Grant McFadden

**Affiliations:** 1 Department of Biochemistry and Biophysics, University of California San Francisco, San Francisco, California, United States of America; 2 Department Biological Sciences, University of Notre Dame, Notre Dame, Indiana, United States of America; 3 Department of Molecular Genetics and Molecular Biology, University of Florida, Gainesville, Florida, United States of America

Over the past three years, *PLoS Pathogens* has become a definitive choice of the wider pathogens research community as a place to publish its best work in the field of microbial pathogenesis. True to our vision, we have freely distributed, worldwide, outstanding original articles that significantly advance the understanding of pathogens and how they interact with their host organisms, and it is our goal to continue to do so.

As part of our development, we have decided to expand the content of the journal. Building on the strength of the Opinions and Reviews, which, like our research articles, are well read and well received by the community, we are pleased to introduce Pearls.

Scientific research, like anything else, is subject to “irrational exuberance.” Fashion, fads, and hypes arrive, have their moment, and disappear from view. In the face of this inevitable ebb and flow of focus and attention, how are we to teach students the “lessons that last” or “the facts of a field” while keeping current? Likewise, how can we teach such lessons in the face of the sheer volume of research that is being published in hundreds of journals?

The need is particularly acute in the area of pathogens research as it covers diverse agents—from prions to arthropods—and approaches—from population genetics to X-ray crystallography. Ostensibly, textbooks serve such a purpose, but such volumes are costly to produce and purchase and require near-constant revision. The standard “mini-review” piece tends to focus on making research recently published more accessible, but these can be too narrow or superficial to live much beyond the moment.

The vision of Pearls is different. Our goal is to produce a substantial *collection* of short (1,500 words maximum) educational and highly useful articles that address topics of relevance and importance within the field of pathogens research. We aim to have each Pearl cover a given topic in a way that makes its significance clear and compelling to a general readership while offering accessible and accurate insight at a graduate student–level.

Intentionally, Pearls will be short, limiting the inclusion of details for their own sake to ensure readability and a sharp focus on the fundamentals. Topics will be digested into two formats; the first is the “five important facts about X” (FIFAX) format and the second is a question and answer (Q&A). Some topics are likely to be better addressed by one or the other and authors will decide whether FIFAX or Q&A works better for a given topic.

Creating this new type of educational resource in *PLoS Pathogens* could not succeed without the enthusiasm and participation (translation: willingness to contribute!) of the scientific community. The response to our invitations to contribute Pearls has been overwhelmingly positive. Numerous experts are preparing these teaching tools, which will be available in the months to come. In this issue, the Pearl is “Virus and Host Determinants of West Nile Virus Pathogenesis” by Michael S. Diamond, which offers a lucid and succinct view of how a virus that entered North America recently avoids elimination by the immune system. This contribution also illustrates mechanistic aspects of the host–pathogen interaction that need to be understood more fully to make possible effective therapeutic strategies for combating an infectious agent capable of serious harm.

Already a leading open-access publisher of new research results, comprehensive reviews, and insightful commentaries in the pathogens community, *PLoS Pathogens* now strives to lead in developing a different kind of scientific writing that, when practically applied, will serve the scientific community in a new way—one that we envision will continually educate as we learn from the front lines of research.

